# Sciatic Nerve Injection Palsy in Children

**Published:** 2016

**Authors:** Viraat Harsh, Romy B. CHENGAZHACHERRIL, Kanika SHARMA, Piyush KALAKOTI, Utkarsh GUPTA, Waqar AHMAD, Anil KUMAR

**Affiliations:** 1 Neurosurgery, Rajendra Institute of Medical Sciences, Ranchi, Jharkhand 834009, India; 2 Madurai Medical College, Madurai, Tamil Nadu 625020 India; 3 Neurosurgery, Louisiana State University Health Sciences Center, Shreveport, LA 71101, United States; 4 Rajendra Institute of Medical Sciences, Ranchi, Jharkhand 834009, India

**Dear Editor-in-Chief**

We read with interest the article by Toopchizadeh et al.(1) reporting outcomes in pediatric patients with Sciatic Nerve Injection injury (SNII) following gluteal injection. Despite the commendable efforts of the authors in long-term monitoring of outcomes in these select cohort of patients with SNII using appropriate electro-diagnostic studies, supplemented use of advanced imaging techniques such as Magnetic Resonance (MR) Neurography to note for structural changes, and diffusion tensor tractography (DTT) for functional altercation, if at all, would have been worth exploring, especially in a setting where the utility of the former in pediatric patients may be limited by poor tolerance (2). The fractional anisotropy (FA) value, a parameter of DTT, is often considered a predictor of functional improvement and a prognostic indicator of nerve injury (3, 4).

The authors plausibly attribute to the thickness of subcutaneous tissue and gluteal musculature as predisposing factors for SNII in pediatric age group. Other pertinent etiologic factors warranting consideration include but are not limited to needle angulation, site of injection, level of training, and patient posturing.

Toopchizadeh et al. meticulously describe known factors such as chemical neurotoxicity, neural ischemia, allergic neuritis, anatomic variations, external scar formation, free radical damage as contributing factors to SNII in a context where injection delivery technique is apropos. Interestingly, other likely factors to impact degree of nerve injury constitute injection pressure, vehicle of suspension, choice of needle and its length, and volume of the injectable (5). Green smith et al. (6) highlight that high-pressure injections (>11psi) cause severe nerve bundle injury as compared to low-pressure injections. Erroneous practices often encountered while administering intramuscular injections amongst healthcare providers, and preventive measures to minimize them are well depicted by Barry and colleagues (7). Interestingly, all patients in the study were managed conservatively via physiotherapy and splints. Villarejo et al. (8) demonstrated excellent results following surgical intervention within 3-6 months of nerve injury. 

Tailoring surgical intervention for cases with minimal or no improvement as noted in clinical assessments or electrophysiologic studies after 3-6 months would have been appropriate. Surgical exploration along with nerve action potential (NAP) recording can be considered as a viable option even in cases with no significant recovery following 3-6 months of conservative management (9, 10). The authors suggest that the prognosis depends on the severity of the primary lesion, however other contributing factors including, but not limited to, are the division involved, level of injury, age of the lesion, timing of repair, patient’s comorbid index (11), and presence of CMAP in the Extensor Digitorum Brevis. Bay suggested that in cases where pain is the only symptom, SNII is fully reversible and the course of recovery may be influenced by psychological factors as well (12).

Considering the disabling impact of SNII on quality of life (QoL), we recommend preventive measures aimed to at decreasing the incidence of SNII in children.

Systematic and schematic training of healthcare providers with respect to the site of gluteal injection (anterolateral), needle angulation for intramuscular route, adequate pressure for flushing the injectable, and prompt identification of possible complications following gluteal injection and measures to mitigate them.
